# Monitoring HOTTIP levels on extracellular vesicles for predicting recurrence in surgical non-small cell lung cancer patients

**DOI:** 10.1016/j.tranon.2021.101144

**Published:** 2021-06-07

**Authors:** Bing Han, Ramón María Marrades, Nuria Viñolas, Yangyi He, Jordi Canals, Tania Díaz, Laureano Molins, Daniel Martinez, Jorge Moisés, David Sánchez, Marc Boada, Melissa Acosta-Plasencia, Coralí Cros-Font, Mariano Monzo, Alfons Navarro

**Affiliations:** a Molecular Oncology and Embryology Laboratory, Human Anatomy Unit, Faculty of Medicine and Health Sciences, University of Barcelona, IDIBAPS, Casanova 143, 08036 Barcelona, Spain; b Thoracic Oncology Unit, Hospital Clinic, Barcelona; c Department of Pneumology, Institut Clínic Respiratori (ICR), Hospital Clínic de Barcelona, University of Barcelona, IDIBAPS, CIBER Enfermedades Respiratorias (CIBERES), 08036 Barcelona, Spain; d Department of Medical Oncology, Institut Clínic de Malalties Hemato-Oncològiques (ICMHO). Hospital Clínic de Barcelona, University of Barcelona, IDIBAPS, 08036 Barcelona, Spain; e School of Basic Medical Sciences, Chengdu University, 610106 Chengdu, China; f Department of Thoracic Surgery, Hospital Clínic de Barcelona, University of Barcelona, 08036 Barcelona, Spain; g Department of Pathology, Hospital Clínic de Barcelona, University of Barcelona, 08036 Barcelona, Spain

**Keywords:** NSCLC, Extracellular vesicles, HOTTIP, lncRNA, liquid biopsy

## Abstract

In resected non-small cell lung cancer (NSCLC), postsurgical recurrence is the major factor affecting long-term survival. The identification of biomarkers in extracellular vesicles (EV) obtained from serial blood samples after surgery could enhance early detection of relapse and improve NSCLC outcome. Since EV cargo contains long non-coding RNAs (lncRNAs), we aimed to analyze whether the oncogenic lncRNA HOTTIP, which higher expression in tumor tissue was related to worse outcome in NSCLC, could be detected in EV from NSCLC patients and serve as recurrence biomarker. After purification of EVs by ultracentrifugation in 52 serial samples from 18 NSCLC patients, RNA was isolated and HOTTIP was quantified by Real time PCR. We observed that patients that relapsed after surgery displayed increased postsurgical EV HOTTIP levels in comparison with presurgical levels. In the relapsed patients with several samples available between surgery and relapse, we observed an increment in the EV HOTTIP levels when approaching to relapse, which indicated its potential utility for monitoring disease recurrence. When we focused in EV HOTTIP levels in the first post-surgical sample, we observed that the detection of an increment of the expression levels in comparison to presurgical sample, predicted recurrence with high sensitivity (85.7%) and specificity (90.9%) and that patients had shorter time to relapse and shorter overall survival. In conclusion, our pilot study showed that EV HOTTIP is a potential biomarker for monitoring disease recurrence after surgery in NSCLC.

## Highlights

• EV HOTTIP analysis during post-surgical follow-up allows early recurrence detection.
• Detection of an increment of EV HOTTIP level in first post-surgical sample predicts recurrence with a sensitivity of 87.5% and specificity of 90.9%.
• Detection of an increment of EV HOTTIP level in first post-surgical sample predicts worse patient outcome.
• EV HOTTIP could be considered as a follow-up biomarker for monitoring recurrence in NSCLC.

## Introduction

The GLOBOCAN2020 reported that lung cancer accounts for 11.4% of total cancer diagnoses and it is still the leading cause of death due to oncological disease in both sexes [1]. Non-small cell lung cancer (NSCLC) is the most frequent type (85% of cases) and is divided mainly into two histological groups, adenocarcinoma and squamous cell carcinoma [2]. The treatment of patients diagnosed with NSCLC depends on the stage of the disease, and in the case of patients clinically diagnosed with stage I-II, the first therapeutical option is usually the surgical resection of the tumor. Although resected patients are considered disease-free after surgery, its prognosis is still very unfavorable with a 5-year survival of 60–80% [3], [4], [5]due to high relapse rates of around 40% [6]. Therefore it is crucial to find prognostic biomarkers to detect patients at high risk of relapse after surgery.

Prognostic biomarkers to classify patients into risk groups have been evaluated by means of tumor tissue analysis [7] and by analyzing the patient's blood, the so-called liquid biopsy [8,9] that includes between others the study of circulating tumor cells, the circulating free DNA/RNA or more recently the study of extracellular vesicles (EV) [10].

Small EV are spherical structures of 30–150 nanometers (nm), consisting of a bilipid membrane with various proteins attached. Inside the small EV group we find exosomes, which origin is associated to mutivesicular bodies [11]. Exosomes are involved in several biological functions, including cross-talk between cells, tumorigenesis, drug resistance, angiogenesis promotion and metastasis. The cargo of the exosomes is enriched in non-coding RNAs, including long non-coding RNAs [12]. LncRNAs is a very heterogeneous group that have been shown to play a relevant role in tumor development and metastasis althought most of them have not been functionally characterized yet [13]. Their high specificity and the fact that they can be found inside tumor released exosomes make them an interesting source of biomarkers. Uptodate few exosomal lncRNAs have been related to tumor prognosis [14]. Some examples in NSCLC includes MALAT1 or lincRNA-p21. High levels of exosomal MALAT1 were related to increased cell proliferation and increased migration [15] and high exosomal lincRNAp21 levels were related to shorter time to relapse (TTR) and shorter (OS) through angiogenesis regulation [16].

HOTTIP is an oncogenic lncRNA related to HOXA locus [17] that has been related to patient prognosis in several tumors [18]. In a previous work of our group, we showed that higher tumor HOTTIP levels correlated with shorter TTR and OS in early stage NSCLC patients [19]. In this line, we decided to perform a pilot study to evaluate whether EV HOTTIP levels could be used as prognostic biomarker. We hypothesized that lung tumors, which overexpress HOTTIP lncRNA, could generate and release small EV charged with lncRNA HOTTIP and their analysis and quantification in peripheral blood could serve to identify patients at high risk of relapse after surgery and to monitorize minimal residual disease for early detection of postoperative recurrence of NSCLC.

## Materials and methods

### Samples

Fifty-two serial blood samples (4 mL in EDTA tube) from peripheral vein were obtained before and after surgery at different times to predict relapse. The time points used after surgery coincided with the postsurgical follow up visits of each patient, which also depends on tumor stage. In all patients at least pre-surgery and first post-surgery sample are available before relapse. Plasma obtained by centrifugation was stored at −80 °C until processing. Written informed consent was obtained from each participant in accordance with the Declaration of Helsinki and the study was approved by the Clinical Research Ethics Committee of the Hospital Clínic de Barcelona (project approval number HCB/2017/1052).

### Extracellular vesicle isolation, RNA extraction and gene expression analysis

EVs from patient samples were isolated and characterized as previously described [19,20]using 200 μL of plasma and ultracentrifugation technique in a Sorvall MX Plus Micro-Ultracentrifuge with the S140AT rotor. Briefly, plasma samples were defrosted on ice and to eliminate potential cellular debris 3 sequential centrifugation steps at 4 °C were performed: 1) 300 G 5 min; 2) 2500 G 20 min; and 3) 10,000 G 30 min. The pellets obtained after each centrifugation step were discarded. Next, the samples were subjected to ultracentrifugation at 100,000 G for 2 h. Then the pellet was washed with DPBS and ultracentrifuged again at 100,000 G for 1 h. After this step, the supernatant was discarded and the pellet was resuspended with 750 μl of Qiazol (Qiagen) supplemented with MS2 RNA (Roche) for RNA isolation. Once resuspended, RNA extraction was completed using miRNAeasy mini kit (Qiagen) according to the manufacturer's instructions. The High-Capacity cDNA Reverse Transcription Kit® (Applied Biosystems) was used to obtain cDNA using 250 ng of total RNA. Relative expression was determined by real-time PCR using the 7500 Real Time PCR device (Applied Biosystems). TaqMan assays (Life Technologies) were used to quantify HOTTIP (Hs00955374_s1) levels in a 7500 Real Time PCR device (Applied Biosystems). 18S (Hs99999901_s1) was used as endogenous control and relative quantification was calculated using 2^−ΔΔCt^.

### Statistical analyses

TTR was defined as the time from surgery to recurrence or last follow-up. OS was defined as the time from surgery to the date of death by any cause or last follow-up. Kaplan-Meier curves for TTR and OS were generated and compared by means of a log-rank test. All factors with *p* < 0.1 in the univariate analysis were included in the Cox multivariate regression analyses for TTR and OS. Paired or non-paired t-tests were used for comparisons between two groups and ANOVA for more than 2 groups comparisons. All statistical analyses were performed using GraphPad Prism 8.4.3, PASW Statistics 18 (SPSS Inc.) and R v2.8.1.

## Results

### Patients

The study included 18 stage I-IIIa NSCLC patients who underwent complete surgical resection in Hospital Clínic of Barcelona. The mean patient age was 65 years (range, 39–83) and 83.3% of patients were male. Six of the seven stage III patients where diagnosed as stage III after surgery when lymph nodes where studied by the pathologist and for this reason only one stage III received neoadjuvant treatment (P11). Eleven patients (61.1%) had received adjuvant chemotherapy after surgery. Median follow-up time was 55.37 months (IQR: 53.03–65.70), during follow-up, a total of seven patients relapsed, six of whom died. The main clinic-pathological characteristics of the patients are summarized in Table 1 and the survival and status at last follow up for each patient is included in Table 2.

**Table 1:** Summary of the main clinical characteristics of the 18 NSCLC patients included in the study. ECOG PS, Eastern Cooperative Oncology Group performance status.

Characteristics	Value	N=18 N (%)
Sex	Male	15 (83.3)
	Female	3 (16.7)
Age, years	Mean (Range)	65 (39-83)
	<=65	10 (55.6)
	>65	8 (44.4)
ECOG PS	0	1 (5.6)
	1	17 (94.4)
pT	1	5 (27.8)
	2	11 (61.1)
	3	2 (11.1)
Lymph node status (pN)	Negative	7 (38.9)
	Positive	11 (61.1)
pTNM Stage	I	5 (27.8)
	II	6 (33.3)
	III	7 (38.9)
Histology	Adenocarcinoma	7 (38.9)
	Squamous cell carcinoma	11 (61.1)
Smoking history	Current smoker	10 (55.6)
	Former smoker	8 (44.4)
Type of surgery	Lobectomy	15 (83.3)
	Pneumonectomy	1(5.6)
	Bilobectomy	2 (11.1)
Adjuvant treatment	No	7 (38.9)
	Yes	11 (61.1)
Relapse	No	11 (61.1)
	Yes	7 (38.9)

**Table 2:** Stage, treatment received and survival and status at last follow up for each patient included in the study.

Patient	pTNM	Neoadjuvant Treatment (Yes/No)	Adjuvant Treatment	Relapse	TTR (months)	Last follow-up Status	Follow-up (months)
P1	Ⅰ	No	No	No	–	Alive	50.03
P2	Ⅲ	No	Chemotherapy	No	–	Alive	50.07
P3	Ⅲ	No	Chemotherapy + Radiotherapy	No	–	Alive	66.87
P4	Ⅰ	No	No	Yes	8.33	Death	28.63
P5	Ⅰ	No	No	No	–	Alive	22.80
P6	Ⅱ	No	Chemotherapy	Yes	33.33	Death	43,93
P7	Ⅱ	No	No	No	–	Alive	55.37
P8	Ⅱ	No	Chemotherapy	No	–	Alive	55.43
P9	Ⅲ	No	Chemotherapy + Radiotherapy	Yes	7.07	Death	13.10
P10	Ⅲ	No	No	Yes	9.70	Death	11.83
P11	Ⅲ	Yes	Radiotherapy	Yes	5.17	Death	5.60
P12	Ⅱ	No	Chemotherapy	No	–	Alive	65.00
P13	Ⅱ	No	No	Yes	18.93	Alive	53.73
P14	Ⅱ	No	Chemotherapy	No	–	Alive	53.03
P15	Ⅱ	No	Chemotherapy	No	–	Alive	55.43
P16	Ⅰ	No	No	Yes	3.13	Death	5.53
P17	Ⅲ	No	Chemotherapy + Radiotherapy	No	–	Alive	60.33
P18	Ⅲ	No	Chemotherapy	No	–	Alive	70.53

### Tracking EV HOTTIP levels in serial plasma samples predicts relapse

**Fig. 1:**
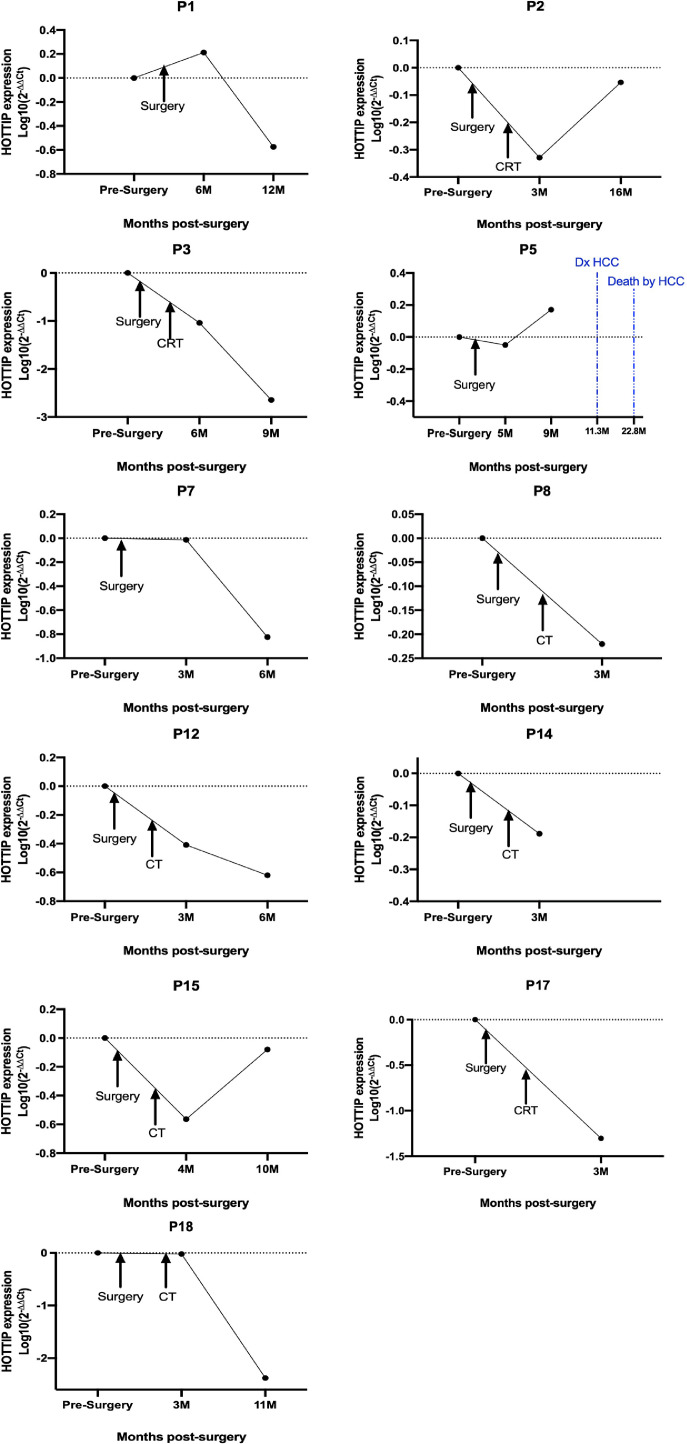
EV HOTTIP tracking in 11 patients who were disease-free after surgery and that not showed relapse at last follow-up. CT, chemotherapy; CRT, chemoradiotherapy; M, months; HCC, hepatocellular carcinoma.

**Fig. 2:**
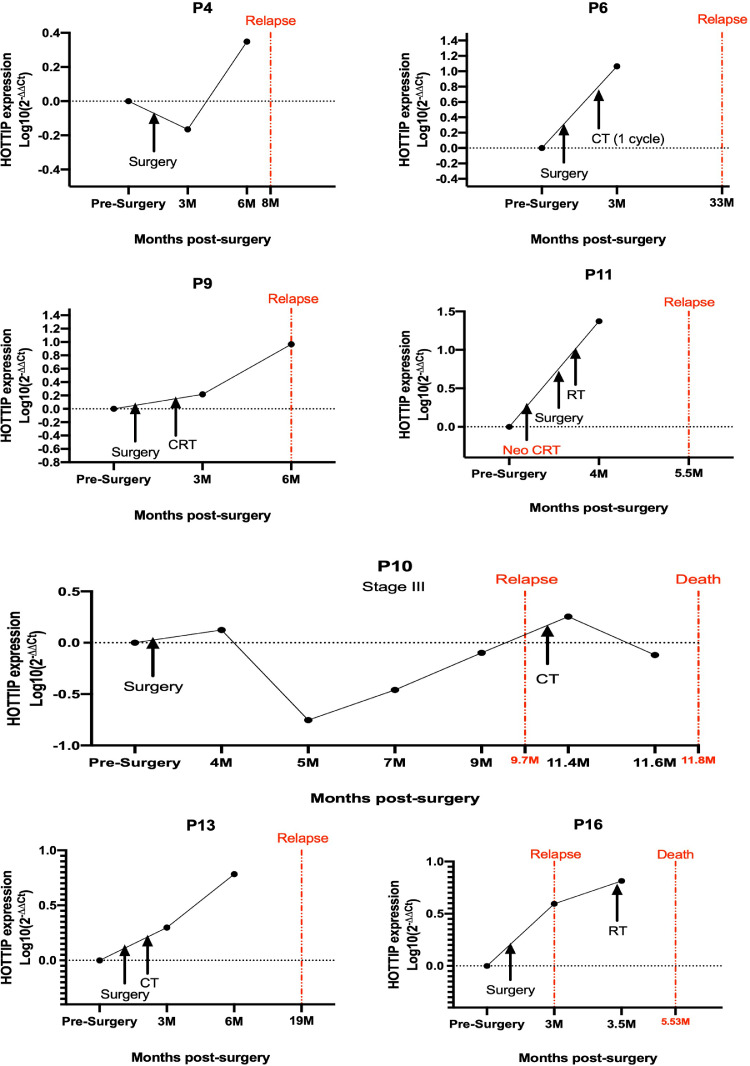
EV HOTTIP levels tracking in 7 patients who were disease-free after surgery but relapsed some months later. CT, chemotherapy; CRT, chemoradiotherapy; M, months; Neo CRT, neoadjuvant chemoradiotherapy.

We investigated the potential utility of HOTTIP quantification in EV for detecting minimal residual disease and relapse prediction. Fifty-two samples from 18 patients were studied including at least the presurgical blood sample and the sample obtained at the first postsurgical visit between 3 and 6 months depending on the disease stage (Figs. 1,2). In the eleven patients that not relapsed after surgery (Fig. 1), we observed that HOTTIP levels in the first post-surgical sample was decreased in 10 of 11. Two of the previous patients (P7 and P18) showed a very small reduction in the EV-HOTTIP levels in the first follow-up visit after surgery (at 3 months), but both showed an important reduction in the second follow-up visit (at 6 and at 11 months, respectively). The unique patient that increased their levels (P1, at 6 months), showed an important reduction in the next follow-up visit (at 12 months). One patient (P5) despite showed a reduction in the first postsurgical sample (at 5 months), showed an important upregulation in the 2nd follow-up visit (at 9 months). Although this patient not relapsed for the lung tumor, he/she was diagnosed with an Hepatocellular carcinoma 11 months after surgery and died from this new tumor 22 months after lung cancer resection.

Seven patients relapsed after surgery (Fig. 2). In all patients we observed an upregulation of HOTTIP levels in comparison with the presurgical sample in the sample obtained previous to the diagnosis of the relapse. In patients with more than one sample after surgery (P9, P10 and P13) we observed that the levels increased progressively. In patient 4 (P4), the levels were downregulated in the first follow-up visit after surgery (at 3 months), but increased at 6 months and the relapse was diagnosed 8 months after surgery.

### Relapse prediction based on evaluation of EV HOTTIP levels on the first postsurgical sample

**Fig. 3:**
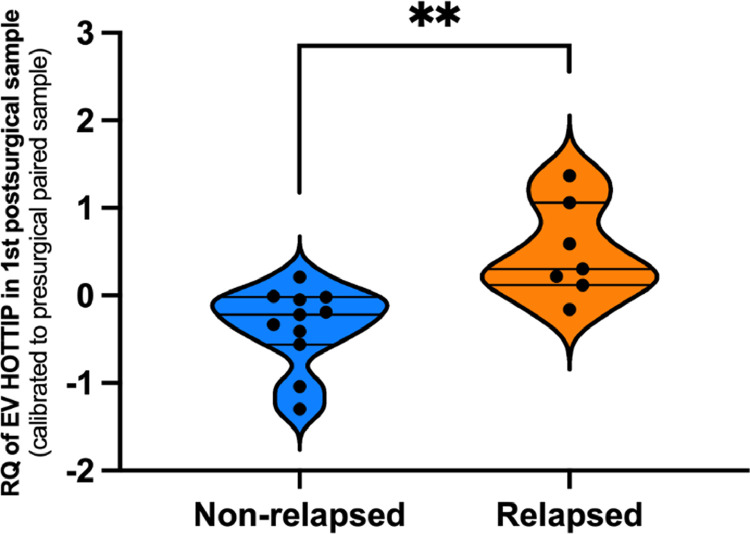
Violin plot showing EV HOTTIP levels in 1st postsurgical sample in relapsed and non-relapsed patients. **p < 0.01.

Since in all patients we had at least the presurgical the first postsurgical follow-up sample, we decided to analyze if with this data we were able to predict relapse. We worked with the EV HOTTIP levels in the first postsurgical sample calibrated to each patient basal presurgical sample (from now on EV HOTTIP in 1st postsurgical sample). We observed that EV HOTTIP in 1st postsurgical sample were significantly higher in relapsed than in non-relapsed patients (*p* = 0.002, Fig. 3).

**Fig. 4:**
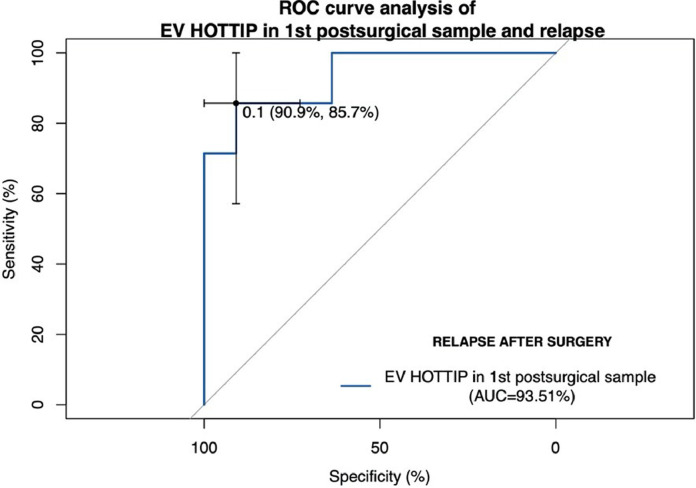
ROC curve analysis of EV HOTTIP ratio values predicting relapse after surgery in NSCLC patients. The best threshold identified was 0.0552 which was rounded to 0.1 in the graph.

Then we decided to evaluate whether EV HOTTIP in 1st postsurgical sample could be used to predict relapse after surgery using ROC curves analysis. The area under the curve (AUC) value according EV HOTTIP in 1st postsurgical sample was 0.935 (95% confidence interval (CI), 0.819–1.0, *p* = 0.002) with a sensitivity of 87.5% and specificity of 90.9% in distinguishing patients who relapse after surgery in its best threshold (0.0552) (Fig. 4). Using 0.0552 as a threshold, the negative predictive value was 90.9%, and the positive predictive value was 85.7%.

### Prognostic impact of EV HOTTIP levels in 1st postsurgical sample

**Fig. 5:**
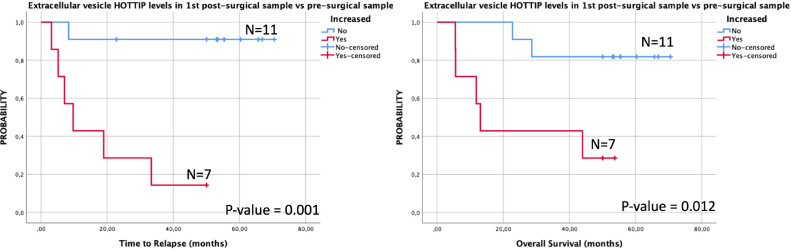
Kaplan–Meier survival analysis showing the different prognosis for TTR (A) and OS (B) between patients with increased vs not increased levels of EV HOTTIP after surgery.

To analyze the prognostic impact of the EV HOTTIP levels in 1st postsurgical sample we divided the patients in two groups according the observed trend in the EV HOTTIP expression in the 1st postsurgical sample in comparsion with its own presurgical sample. Then we grouped patients in patients which expression increased and patients with non increased expression. Using this classification, we observed that patients where EV HOTTIP levels increased after surgery had shorter TTR (9.7 months vs not reached; *p* = 0.001) and shorter OS (13.1 months vs not reached; *p* = 0.012) than patients that had no change or reduced their levels (Fig. 5).

### Multivariate analysis showed that EV HOTTIP in 1st postsurgical sample is an independent prognsotic marker of TTR and OS

EV HOTTIP levels in 1st postsurgical sample, disease stage and adjuvant treatment, were included in the multivariate analyses. High EV HOTTIP levels in 1st postsurgical sample emerged as an independent prognostic marker for shorter TTR (Hazard ratio [HR]: 6.621; 95% confidence interval [CI]: 1.599–27.415; *p* = 0.009) and shorter OS (HR: 6.586; 95% CI: 1.331–32.595; *p* = 0.021). Adjuvant treatment administration emerged also an independent factor for OS (HR: 0.106; 95% CI: 0.018–0.641; *p* = 0.015).

## Discussion

Postoperative recurrence is the major factor affecting the long-term survival of resected NSCLC patients [21]. The postoperative follow-up should ideally include the analysis of molecular markers enhancing early detection of recurrence and metastasis allowing the identification of patients candidates for an aggressive treatment strategy [22,23]. In the context of stage I NSCLC patients, who not receive adjuvant treatment after surgery this could means to detect patients with a more aggressive disease that could benefit from adjuvant chemotherapy treatment. However, at present, effective testing methods for postoperative follow-up remain to be established, and guidelines recommended by major organizations in western countries differ considerably [22,23]. The aim of our study was to find an effective and non-invasive method to predict and monitor postoperative recurrence by examining EV HOTTIP levels in resected NSCLC patients treated and followed in our center after curative surgery.

To our knowledge, we have provided the first evidence that EV HOTTIP levels are associated with NSCLC recurrence and it is a useful tool to monitor patient progression after surgery. We observed that patients that relapsed some months after surgery displayed an increase of the postsurgical EV HOTTIP levels in comparison with presurgical basal levels. In the patients with several samples available at different times after surgery and before clinical detection of relapse, we could observe an ascendent trend in the EV HOTTIP levels, which indicated its potential utility for monitoring disease evolution. Interestingly, we detected one patient (P5) that not relapsed but the increase in the EV HOTTIP levels were linked to the diagnosis of Hepatocellular carcinoma 11 months after lung tumor resection. Despite of its diffciult to make conclusions only with one patient, we could speculate that probably EV HOTTIP could be used to monitor disease evolution in other malignancies.

When we focused in the utility of the analysis of EV HOTTIP levels only in the first postsurgical sample available, which was usually between 3 and 6 months after surgery (coinciding with the patient postsurgical follow-up visit, which is mainly depending on the patient stage), we observed that the detection of an increment of the expression levels in comparison with the basal presurgical sample was able to predict recurrence with high sensitivity (85.7%) and specificity (90.9%). In this line the patients which EV HOTTIP levels increased in the first postsurgical sample had shorter TTR and shorter OS than patients with no change or with lower levels. Finally, the multivariate analysis showed that EV HOTTIP levels in first postsurgical sample calibrated to presurgical sample is an independent marker for both TTR and OS. Although to our knowledge no other studies are available analyzing in serial blood samples the clinical potential of tracking EV HOTTIP levels, it has been shown in other tumors that EV HOTTIP levels can act as diagnostic and prognostic biomarker. In gastric cancer high levels of EV HOTTIP correlated with poor OS and had a higher diagnostic capability than traditional clinical markers such as CEA levels [24]. In contrast, in colorectal cancer lower EV HOTTIP levels were associated with decreased OS, but in the same line they observed that low tumor levels were also related to worse postsurgical prognosis of these patients [25]. Interestingly, Wu F. et al. [26] showed that HOTTIP levels can also be detected in EV purified from bronquioalveolar lavage from NSCLC patients, which indicates that the analysis of HOTTIP levels in this type of samples could also have utility for diagnosis or prognosis analysis in NSCLC patients, but the authors not analyzed specifically the utility of HOTTIP as biomarker is this medium.

The role of HOTTIP in tumor cells have been widely studied showing that HOTTIP acts mainly as an oncogene in the majority of cancers [18]. In most tumors HOTTIP is upregulated in comparison to its normal counterpart and its primary oncogenic role is related to modulation of HOXA genes expression through histone methylation [27]. Moreover, it has been described that HOTTIP levels are related to smoking history in tumor tissue [19]. In the present work all included patients were either current smokers (*n* = 10) or former smokers (*n* = 8). We analyzed (data not shown) whether significant differences could be observed between both groups according to smoking status in both presurgical blood sample and in the first post-surgical sample, and no significant differences were observed. Taking in account this results and the fact that in our previous paper we did not observed significant differences between current and formers smokers, that corresponds to most of the patients in NSCLC, we consider that the smoking history of the patients will not affect the use of HOTTIP as prognostic tool. Moreover, the fact that we evaluated HOTTIP levels in relation to baseline sample of each patient (presurgical sample) we consider that we are correcting potential biases that could emerge in relation with the smoking history or other clinical characteristics of the patients.

Additional oncogenic mechanisms have been described in different tumors. In NSCLC, HOTTIP has been described to promote proliferation and migration through HOXA13 regulation [28] and also drug resistance by regulation of the AKT pathway [29]. Interestingly, it has been shown that in NSCLC cells HOTTIP become upregulated in hypoxic conditions and promotes glycolysis by regulation of HMGB3 gene indirectly through sponging miR-615–3p [30]. In the same line, but in an atherosclerosis model it has been shown that HOTTIP regulated endothelial cell proliferation and migration by induction of B-catenin expression and modulation of c-Myc pathway [31]. Taken together all this information we can speculate that tumor cells can modulate endothelial cell behavior in hipoxic conditions by releasing EV containing high levels of HOTTIP and this could enhance the metastasis process. This potential mechanism of action have been observed previously in NSCLC with other EV lncRNAs such as lincRNA-p21 [16].

In summary, we have showed in this pilot study that EV HOTTIP can be used as a liquid biopsy biomarker to monitor disease recurrence after NSCLC surgery. Its analysis in serial samples after surgery could be of potential utility to predict whether patients will develop cancer recurrence or metastasis within one year after surgery. However, the present study has several limitations including the low number of patients enrolled in the study or the fact that relative quantification has been performed for HOTTIP analysis. In relation to the low number of patients, a prospective analysis including more patients may be warranted to validate the potential utility of this follow-up biomarker. The most recent clinical guidelines analyzing the available tools for NSCLC surveillance after curative surgery, not recommends the use of blood biomarkers because not enough experimental evidences and studies in large cohorts support their utility, and therefore the image techniques are yet the most extended method for monitoring recurrence [32]. Blood biomarkers are only recommended in the context of clinical trials indicating that we are far from translate from bench to clinic the use of these type of biomarkers, but the validation in additional cohorts of results like the ones included in the present paper can be the first step to accelerate the inclusion of blood biomarkers in routine clinical use. By another hand, the other limitation of the present study was related to the quantification method used. The use of absolute quantification without the use of a reference gene may enhance the clinical use of HOTTIP or other identified EV-associated lncRNAs in the clinic. The more extensive use of digital PCR could solve this limitation in a forseable future. Although, these limitations, we think that the finding are significative since no prior research have been performed in this topic and opens a door to further analyze the potential utility for monitoring minimal residual disesease in resected NSCLC patients using EV lncRNAs. Functional studies to fully understand the role of EV HOTTIP will also increase the validity of this new potential biomarker. We believe that in the near future we will see not only the use of EV HOTTIP levels as recurrence tracking marker in NSCLC, but also in other tumors and the identification of more EV lncRNAs with clinical utility.

## Conclusions

In conclusion, we identified EV HOTTIP as a biomarker for predicting postoperative recurrence in patients with NSCLC. Our study has shown that when the expression levels of EV HOTTIP increase during the postsurgical follow up, the patient is highly likely to have cancer recurrence or metastasis. Therefore, EV HOTTIP should be considered as a promising key factor in predicting postoperative risk of NSCLC recurrence. Considering the diversity of exosome cargos and their important role, more studies should be carried out with these molecules in the future to detect the risk of recurrence after NSCLC surgery.

## Declaration of Competing Interest

The authors declare that they have no known competing financial interests or personal relationships that could have appeared to influence the work reported in this paper.
